# Towards a resolution of some outstanding issues in transitive research: An empirical test on middle childhood

**DOI:** 10.3758/s13420-020-00440-7

**Published:** 2020-08-12

**Authors:** Barlow C. Wright

**Affiliations:** Department of Psychology, Nottingham Trent University, 50 Shakespeare Street, Nottingham, NG1 4FQ UK

**Keywords:** Children’s reasoning, Dual process, Memory, Training, Transitive inference

## Abstract

Transitive Inference (deduce B > D from B > C and C > D) can help us to understand other areas of sociocognitive development. Across three experiments, learning, memory, and the validity of two transitive paradigms were investigated. In Experiment 1 (*N* = 121), 7-year-olds completed a three-term nontraining task or a five-term task requiring extensive-training. Performance was superior on the three-term task. Experiment 2 presented 5–10-year-olds with a new five-term task, increasing learning opportunities without lengthening training (*N* = 71). Inferences improved, suggesting children can learn five-term series rapidly. Regarding memory, the minor (CD) premise was the best predictor of BD-inferential performance in both task-types. However, tasks exhibited different profiles according to associations between the major (BC) premise and BD inference, correlations between the premises, and the role of age. Experiment 3 (*N* = 227) helped rule out the possible objection that the above findings simply stemmed from three-term tasks with real objects being easier to solve than computer-tasks. It also confirmed that, unlike for five-term task (Experiments 1 & 2), inferences on three-term tasks improve with age, whether the age range is wide (Experiment 3) or narrow (Experiment 2). I conclude that the tasks indexed different routes within a dual-process conception of transitive reasoning: The five-term tasks indexes Type 1 (associative) processing, and the three-term task indexes Type 2 (analytic) processing. As well as demonstrating that both tasks are perfectly valid, these findings open up opportunities to use transitive tasks for educability, to investigate the role of transitivity in other domains of reasoning, and potentially to benefit the lived experiences of persons with developmental issues.

Transitive reasoning is investigated using tasks of linear syllogisms, dominance hierarchies, relational integration, and learning of ordered sets (Dai, 2017; Holcomb, Stromer, & Mackay, 1997; Ricco & Overton, 2011). When making a transitive inference, the reasoner draws on two pieces of information—here denoted as premise BC and premise CD (reasons for this denotation will become clear below). The premises must be describable in terms of a common relational term (e.g., “nicer than”) and must overlap at a common point (here, at item C). From these, the reasoner actively deduces the results of a third possible but latent comparison (here, the B?D comparison) without needing to experience that comparison perceptually in the real world (Bara, Bucciarelli, & Lombardo, 2010).

Transitive tasks have contributed much to developmental psychology—for example, regarding mathematical development, dyscalculia, fluid intelligence, hemispheric specialization, and attention-deficit/hyperactivity disorder (Brunamonti et al., 2017; Castle & Needham, 2007; De Neys & Vanderputte, 2011; Morsanyi, Devine, Nobes & Szucs, 2013; Schwartz, Epinat-Duclos, Leone, Poisson, & Prado, 2020; van Duyne & Sass, 1979). Given the well-accepted applications of transitive tasks for so many purposes, it is surprising to note that one of the most fundamental debates, concerning transitive tasks, has not yet been resolved. This debate, perhaps best captured by Bryant (1998), concerns the relative validity of two alternative ways of assessing transitive reasoning. In brief, one way is to use five-term tasks and another is to use three-term tasks; although, as will become clear below, the differences go well beyond the variation in number of items.

Three-term tasks contain the most basic linear syllogistic structure: Provide B > C and C > D; infer B > D (Morsanyi et al., 2013; cf. Sternberg, 1980; Wright, Robertson, & Hadfield, 2011). Using these, Piaget and Inhelder (1956/1967) reported that children develop transitive competencies from around 7 or 8 years of age (see also Ameel, Verschueren, & Schaeken, 2007; Dai, 2017; Markovits, & Dumas, 1999; Verweij, Sijtsma, & Koops, 1996). However, Bryant (1998) raised three key issues against these tasks. First, three-term tasks were claimed as inherently unable to assess deduction, whereas other transitive tasks (see below) routinely index deduction. Of note, Bryant’s lab used three-term tasks (e.g., Bryant & Kopytynska, 1976) or extrapolations of three-term subseries from larger series (e.g., Pears & Bryant, 1990). This implies acceptance that in certain circumstances, three-term tasks can test the target transitive competence (Bara et al., 2010; cf. Stevens, 1951).

Second, using only three terms creates the risk that children will routinely use the unique label given to the largest item rather than any kind of inference, logical or not, to reach the transitive inference (e.g., parroting/categorical labelling; Wright, 2001). Therefore, three-term tasks, by virtue of having only three terms, can never validly index transitive inference (Bryant, 1998). Yet no theorist espousing that view supports it with direct empirical evidence. In the absence of such evidence, it is scientifically prudent to remain open to the alternative view that there might be many ways of generating transitive responses (Ameel et al., 2007; MacLean, Merritt, & Brannon, 2008; Piaget, Grize, Szeminska, & Vinh Bang, 1968/1977; Premack, 2007).

Third, children may fail three-term tasks because of poor memory rather than because of not possessing the logical capacity needed for transitive inference. We can address this potential issue by ensuring that the child remembers the premise information before going ahead with testing for the inferential response. However, although Bryant’s lab identified this potential shortcoming, they never modified the three-term task to deal with it (e.g., it was not applied in Bryant & Kopytynska’s, 1976, use of the standard three-term task). Rather than modify three-term tasks to avoid the above issues, Bryant and Trabasso (1971) popularized a task based around five or more terms, employing repeated training on a minimum of four premises (Amd & Roche, 2016; Berens & Hayes, 2007; Holcomb et al., 1997; Kumaran & Ludwig, 2013; Titone, Ditman, Holzman, Eichenbaum, & Levy, 2004).

Several issues arise regarding five-term tasks. The first is about which item-pair is equivalent to the critical pair in the three-term task. One can understand this by starting with the denotation of the three-term task as A > B plus B > C leads to A > C. In the five-term task there are two additional premise pairs, C > D and D > E. So, on the surface it might look as though the equivalent critical comparison on the five-term task is either B versus D or A versus E. This causes little ambiguity in research relying only on one task type or the other. But if both task types need to be directly compared, the old denotation can easily cause confusion. In that event, the most prudent thing to do is to give the pairs that are to be compared equivalent names across the two paradigms. The most straightforward way of achieving this equivalence is to relabel the items in the three-term task as B, C, and D.

The second issue is about comparability of what each task theoretically measures. Whilst the five-term task avoided end items being used as an index of deduction, it introduced a new problem as serious as the one it claimed to solve. Namely, it replaced two premise pairs (BC & CD) and one inference (BD) on the three-term task, with at least four premise pairs (AB, BC, CD, & DE) plus six inferential pairs (AC, BD, CE, AD, BE, & AE). Five-term tasks increased the memory load at least two-fold and increased the inferential load at least six-fold. The overall number of paired relationships increased from three pairs on a three-term task to 10 on a five-term task, and for a six-term task it would be an even higher 15 pairs. However, although transitivity of three-term sets can be considered a matter of logic (Bara et al., 2010; Piaget et al., 1968/1977; Stevens, 1951), no theorist offered a logical or mathematical basis for expecting that a five-term task should index the same capacity, the same level of difficulty or the same memory load as does a three-term task. That said, as the Bryant and Trabasso (1971) claim was widely accepted in the developmental, adult, learning difficulties, and comparative psychological literature (Gazes, Lazareva, Bergene, & Hampton, 2014; Holcomb et al., 1997; Stromer, Mackay, Cohen, & Stoddard, 1993), this claim was tested in the present research.

Third, whereas one might need to present premises on a three-term task only once each (Wright & Dowker, 2002), on the five-term task this results in lack of learning the premises (Wright, 2001). Therefore, Bryant and Trabasso (1971) introduced “extensive training” (Yamazaki, 2004). This ensured that premises were learned, and only after this training would participants move to an inference-testing phase (Brunamonti et al., 2017; Kumaran & Ludwig, 2013; Qu, van der Henst, & Dreher, 2017). However, even in some studies with typically developed adults, only 60% of reasoners pass the task after highly extensive training to a criterion of 100% (e.g., Amd & Roche, 2016). This situation contrasts against Stromer et al. (1993), who trained a typically developing 5-year-old child and two adults with mental ages similar to the child participant on two totally independent five-term transitive tasks. When participants were asked to compare B from one task versus D from the other task, B was chosen 100% of the time. This is a systematic strategy, but clearly not a logical one (Wright, 2001). This raises the important question of what bearing amount of training has on the nature of our mental processes, when using the extensive-training paradigm (Amd & Roche, 2016; Wright, 2006a).

Fourth, Bryant and Trabasso (1971) claimed that “all groups learned the initial (direct) comparisons rapidly” (p. 457). This leads us to assume that their criterion level for premise training (90% correct responses) was reached in a single session. With this training, children down to age 4 years did rather well (i.e., at least 78% correct) on the critical BD inference. However, this situation contrasts with Riley and Trabasso (1974), who needed to spend 5 days on training children and so concluded it might not be possible to train children using rapid learning (see also Berens & Hayes, 2007; Gazes et al., 2014; Holcomb et al., 1997; Trabasso, van den Broek, & Suh, 1989). This issue is important to settle. For instance, it might be considered unethical to remove children for more than half an hour’s transitive training in school, as they might be missing out on that day’s teaching in an important subject (Brunamonti et al., 2017; Castle & Needham, 2007; Long & Kamii, 2001; Morsanyi et al., 2013; Rabinowitz, Grant, Howe, & Walsh, 1994).

There are also issues that affect both tasks, such as about relationships between reasoning and memory. Earlier, memory of the premises was intimated to be crucial in generating inferences (Chapman & Lindenberger, 1992). Indeed, several investigators used the product of BC and CD, as a predictor for the BD inference (Bryant & Trabasso, 1971; Wright, 2006a; Wright & Howells, 2008). This contrasts with other studies that have reported the statistical independence of memory for the premises and deducing the inferential comparison (Brainerd & Reyna, 1992). It may be that memory dependence exists on one type of task, but memory independence on the other.

As another issue, it is assumed that series acquisition proceeds from the unmarked end of the series (e.g., larger end; Titone et al., 2004; Wright, 2006a). This predicts that of the two pairs logically required to make the BD inference, BC should be remembered better than CD, and the difference between BC and CD should favour the BC pair. In Bryant and Trabasso (1971) data summary for 4–6-year-olds (Experiment 1), the opposite profile was observed for two of their three groups, with only the 4-year-olds showing a BC advantage (see also Wright & Smailes, 2015). This raises the question of whether there is a CD superiority effect.

A final issue is which of the antecedent premises, if any, drives the BD inferential response. Brainerd and Reyna (1992) report that after highly extended training, the “gist” of the transitive series is represented in memory from the larger end to the smaller end; it follows that in the case of BC, CD, and BD, the larger of the premise pairs should be more closely associated with the BD inference. However, few if any of these important memory issues were explicitly drawn out of the relevant papers. Clearly, the role of memory in transitive responding needs further investigation both with extensive-training tasks and three-term tasks.

## Summary of aims

Using three studies containing different task variants, the first of four main aims was to provide the first direct comparison between extensive-training tasks and three-term tasks. The second aim was to satisfy issues levelled against either the extensive-training task or the three-term task. These include whether categorical effects lead to performance at ceiling, plus the testing of premise memory after the giving of transitive responses. The third aim was to assess additional memory issues, such as whether levels of premise retention and the relationship between premise memory and the inferential response vary with age or across three-term versus extensive-training tasks. The fourth aim was to determine whether it is possible to teach children the premises on a five-term extensive-training task relatively rapidly. I operationalized “rapidly” according to Riley and Trabasso (1974), as needing to remove a child from no more than a single 25–30-minute primary-school lesson such that children both remember the premises well and potentially do well in giving transitive responses.

As one point of agreement between extensive-training advocates and three-term advocates is that children are competent in deductive transitive reasoning by or before they are 7–8-years-old, the present tasks were restricted to groups of mean age 4 to 8 years.

## Experiment 1

### Method for Experiment 1

#### Participants

A total of 121 children participated (*M* = 7.6 years, *SD* = 0.315). There was roughly an equal split of girls and boys in each group. All children were typically developing, with English as their first or only language. Children were allocated to a three-term nontraining task or a five-term extensive-training task (see below for task details). This resulted in 77 children of 7.1 to 7.9 years, assigned to the three-term nontraining task (*M* = 7.5 years, *SD* = 0.180), and 44 children of 7.4 to 8.6 years assigned to the five-term extensive-training task (*M* = 7.9 years, *SD* = 0.327). The variation in sample size for the two respective tasks was due to the nontraining task taking less time to complete than the extensive-training task. Given the quite reasonable constraints on total time allowed by schools, this meant fewer children could be tested on the latter task.

#### Materials

The classical transitive inference task is the three-term task and such tasks can be learnt with as few as one presentation of the premises (Wright & Dowker, 2002). By contrast, five-term tasks typically require highly repeated training on the same premises repeatedly (Wright, 2006a). According to Wright (2001), repeatedly training participants on the same three-term task would lead to direct recollection of the BD inference, rather than this pair being resolved via any kind of reasoning. Therefore, to begin to make the two types of task as equivalent as possible and also to permit the collection of enough BD responses so as to allow parametric statistical analyses, a different three-term series needed to be used, to correspond to each successive round of training and test on the five-term task (four tasks/sessions in each case, excluding practice/familiarization trials).

The materials for the three-term task were based on Wright and Dowker (2002). These were three-dimensional rod-like objects that were either circular or square in cross-sectional area. There were two versions of each shape, based on increasing cross-sectional area (width/diameter = 2cm vs. 4cm). Each of the four sets comprised one item of each of the following colours: red, blue, green, yellow, and white. A three-term series was constructed by selecting three items within a given set.

Octagonal cards painted with each of these colours were also used (width/diameter = 3 cm). In this task, randomized assignments of object to serial order position were used, but once assigned, the ordinal position for each object was fixed.

The extensive-training task used a PC compatible laptop computer with a 17-inch screen. This was a computer-presented version of the Bryant and Trabasso (1971) task. It used the relational dimension of “length”, equivalent to that used in the three-term task. Colours used were the same as the three-term task. To mimic the discrete objects in the three-term task, which each had their own colour and length, once a given mapping of colour onto order had been introduced for a given participant, that mapping was retained for the remainder of the task. A separate dedicated external USB numeric keypad was used to collect responses. White ring reinforcers (which looked like a Polo Mint) were stuck onto the centre of the “4” key and the “6” key, so the child knew which keys to choose from in the experiment.

#### Design

The basic design was between subjects, with the 7-year-olds doing one of the two tasks (three-term task vs. five-term task).

#### Procedure

For this and the other experiments reported here, ethical approval and parental consent were gained prior to testing. Testing took place in a quiet but familiar room within the school. The two tasks make use of seemingly incompatible methodologies (Bryant, 1998), and so the aim here was to make them as similar to each other as possible, via their procedural regimes. Here, both tasks used a practice/familiarization stage, with transitive series different to those used in the tasks proper, followed by discussion not connected with the task, and ending with the task proper.

The procedure for the three-term task began with the child being shown one example of each of the possible shapes and sizes, using four different colours overall. After this familiarization, a practice trial was given. Here, the experimenter held up two objects (e.g., B & C) at a time, about 1.5 m apart. The child indicated which one was longer. The items were now removed from view, and the next item pair (e.g., C with D) was shown in the same way. Next, with all three items now out of view, the child recapped the two pairs of items that had been shown, by reporting their colours and which one had been the longer of each pair. All children reported the item shapes, respective colours, and the size relationships correctly.

The inferential question was asked via a two-alternative forced-choice format using octagonal colour cards. Holding up two cards in random order, I asked, “So if I showed you the object that was this colour and the object that was this colour, which one do you think would be the longer of those two?” Finally, after the child gave an inferential response, memory for the premises was rechecked by using the octagonal cards.

A difference between practice and proper trials was that in the practice trial, the child was encouraged to ask the experimenter if he or she was unsure about any aspect of the procedure and was encouraged to explain his or her thinking (Verweij et al., 1996). Also, in the practice trial, the child was given feedback about whether the premises had been correctly retained in each of the memory phases (before and after the inferential response).

Following the practice/familiarization stage, the child was engaged in a brief discussion for around 2 minutes, to reduce the risk of the practice trial interfering with the task proper (proactive interference). Next, four proper trials were given, using the above learning, test, and memory phases. Each used a different one of the four compositions of item cross-sectional shape (circular or square) and item width/diameter (2 cm or 4 cm).

On the test questions of the proper trials, no feedback was given regarding the correctness of the inference or of the memory pairs. In this way, I adequately addressed an issue raised about memory by Bryant and Trabasso (1971) and Bryant (1998), in so far as poor memory during premise acquisition cannot be blamed for subsequent inferential performance. This also meant that, perhaps for the first time, it was possible to determine whether memory performance remains at 100% after the child has answered the inferential question.

The four trials were ordered randomly. Each child received one mark for each correct answer to test/memory questions, meaning the maximum possible score across BC trials was 4, and likewise for CD and for the BD inference trials. Including briefing, debriefing, practice trial, brief discussion, and proper trials, the three-term procedure took around 15 minutes to administer.

The extensive-training task used two practice/familiarization trials on a single series. These each necessarily contained four premise pairs because the implied series had five items in all. Practice was followed by a discussion as before. The proper trials were then given. Proper trials fixed the amount of training at five introductions to the four premises (training increments), to render this task as comparable as possible with the three-term task whilst giving children the opportunity to learn the premises well (Bryant & Trabasso, 1971). Note, the first learning-test increment was discounted, as during it the child was not yet used to the specific premise or test pairs constituting the transitive series (which was different from the series used for practice).

Before beginning the task proper, a shortened version of it, having only two increments, was used to familiarize the child with the task context and responses required. This meant the child experienced one serial presentation and one random presentation of the premise pairs. This practice was followed by a short discussion of the child’s day to reduce the chance of proactive interference with the following proper task, and then the proper task was given. The practice stage provided a test phase after the serial presentation of the premises, but did not present a test phase after the randomized premises; this was only done in the task proper. However, the first learning-test increment proper served to further familiarise children with the format of the test phases.

Children were instructed verbally about the task and shown how to use the numeric keypad to tell the computer their answers. They sat approximately 60 cm from the screen. In any learning phase, each of the four premises was shown once. Two sticks comprising a premise were gradually revealed from behind a pair of curtains opening upwards and downwards from the middle. The curtains stopped before revealing the entirety of either stick. One stick was to the left of the centre of the screen and the other was to the right. Their position was randomly determined. Once the curtains were stationary, children were asked to press a designated key to indicate the stick they thought was longer. They used the separate numeric keypad to tell the computer this. If children thought the longer stick was on the left of the screen, they pressed the “4” key. If it was on the right, they pressed the “6” key.

Feedback was threefold. The curtains opened further to reveal the full length of each stick so that the child could see whether his or her response had been correct. Simultaneous with this feedback, a high-pitched or low-pitched tone indicated a correct versus incorrect response, and the word “Correct” or “Wrong” was displayed in large text at the bottom of the screen. After a break of up to 3 seconds, the next premise pair was presented in the same way.

Each pair was on screen for as long as required and controlled by participant key presses, which would result in the curtains opening (as described above). The first presentation of the four premises always came in ascending or descending order (Riley & Trabasso, 1974). This direction (ascending vs. descending) was generated randomly by the computer program (Wright, 2006a). Following Moses and Ostreicher (2010), the subsequent four increments each used a newly randomized premise presentation order.

In the task proper, every learning phase was followed by a test phase. In the test phase, each of the four premises was presented along with the six possible inferential pairs. However, no feedback was given: The curtains stopped opening just before they would have revealed the tops and bottoms of the sticks, and no tonal or text feedback was provided. Thus, here, just as with the three-term task, the child was not given feedback about whether a particular decision had been correct or not.

The test increment tested each of the four premises and the six possible inferential pair-wise comparisons a total of four times, to give a total of 40 test trials. The only nonrandom criterion was that all 10 pairs were tested exactly once, before any of them could be tested again. But within each round of testing the order of asking the memory and inferential questions was randomized anew (following Riley & Trabasso, 1974). This gave a maximum possible score of 4 for each respective premise pair and each respective inferential pair, just as for the three-term task.

The data comparable with the three-term task were drawn from only the final test increment; the purpose of the prior learning-test phases was to improve the child’s premise performance as much as possible, given the very reasonable time constraints of the school. From this increment I focused only on the pairs critical to the fairest but most valid test of transitive reasoning capacity (i.e., BC, CD, and the BD inference; see Bryant, 1998; Wright, 2006a). For this task, briefing, debriefing, practice/familiarization, brief discussion, and proper task took around 27 minutes per child.

### Results and discussion for Experiment 1

The critical BCD data of interest were analyzed using parametric tests with an alpha level of 0.05. The inferential (BD) data are presented in Table 1, both as values out of 4 and as percentages to allow direct comparison against previous studies. Table 1 shows that BD performance was relatively good, both on the three-term task and the five-term extensive-training task. However, on the three-term task, performance was 47.9% higher relative to the five-term task (see Table 1 for absolute values).

**Table 1:** Memory and inferential performance for three tasks across Experiments 1 and 2

	Premise pairs (Memory)			Inference
	BC	CD	Both	BD
Experiment 1				
5 term	2.311 (0.129) 58%	2.211 (0.128) 55%	2.261 (0.079) 57%	2.249 (0.171) 56%
3-term	3.321 (0.099) 83%	3.498 (0.098) 87%	3.409 (0.061) 85%	3.052 (0.139) 76%
Both tasks	2.839 (0.077) 71%	2.844 (0.076) 71%	2.841 (0.056) 71%	2.702 (0.097) 65%
Experiment 2				
5 term	2.593 (0.102) 65%	2.618 (0.101) 65%	2.605 (0.063) 65%	2.472 (0.123) 62%
All 3 tasks	2.742 (0.064) 69%	2.776 (0.063) 69%	2.759 (0.039) 69%	2.591 (0.075) 65%

*Note.* Memory and inference scores are given after controlling for age. Figures in parentheses are standard errors of mean scores. Percentages are included for ease of comparison with other research

The overall difference in BD performance between the two tasks was assessed using a one-way analysis of covariance (ANCOVA). Task was the between-subjects factor, having two levels corresponding to the three-term task versus the extensive-training task. Children’s exact ages (in years, to 3 decimal places) and their memory scores were entered as the covariates, to allow assessment of task performance controlling as much as possible for small variations in age and memory (see Moses & Ostreicher, 2010, for similar use of memory scores). The analysis confirmed that the high inferential performance on the three-term task compared with the five-term task was statistically significant, *F*(1, 117) = 9.585, *p* = .004. Age was not a statistically significant covariate, *F*(1, 117) < 1, which was unsurprising given that children had been deliberately selected mainly within a single school year. However, memory was a significant covariate, *F*(1, 117) = 5.084, *p* = .001, and this is considered further later on.

The findings for inferential performance on the extensive-training task are not perplexing if taken in the context of findings from a recent task with adults. Moses and Ostreicher (2010) reported that older adults (i.e., over 60 years old) who are not made explicitly aware of the linear ordering of items (e.g., Item A to Item E) also perform near 50%. It may simply be, then, that here the 7-year-olds given the extensive-training task did not explicitly appreciate the fully transitive nature of the five-term series. For the three-term task, the level found here is consistent with that of Ameel et al. (2007) in a spatial three-term transitive task (they reported 74% at age 8.3 years).

Turning to memory, Table 1 summarizes memory for each of the two premises in turn (BC & CD) as well as the mean of these (constituting the memory covariate in the previous analysis). It shows a tendency for memory in the three-term task to be much better than memory in the extensive-training task. Memory performance was assessed using a two-way mixed-model ANCOVA. The between-subjects factor was task. The second factor was premise, which was the repeated factor, with two levels corresponding to the logical antecedents for the BD inference (i.e., the major premise BC and the minor premise CD). As before, age was entered as a covariate as a precaution. In this analysis, age was not statistically significant as a covariate, *F*(1, 118) < 1. This confirms that memory did not reliably improve with age, across a single school year.

Of main interest, though, the main effect of task was statistically significant, *F*(1, 118) = 58.795, *p* < .001, confirming that the extensive-training task did not lead to as high memory as the three-term task. However, the main effect of premise was not statistically significant, *F*(1, 118) < 1. Any tendency toward a two-way interaction between task and premise did not approach statistical significance, *F*(2, 118) < 1.

## Experiment 2

An interpretation of Experiment 1, is that extensive-training tasks are more difficult to learn and solve than are three-term tasks. According to Wright (2006a), this is partly because the retention of four interlinked premises places greater memory load on the reasoner than does the retention of only two interlinked premises (Perner & Mansbridge, 1983). However, to answer why this is, it is prudent to look for specific factors that might underlie the difference in difficulty. One possibility is that the use of five increments of training for the five-term task, with testing after each increment, fatigued the young participants by the time they got to the final test increment (increment 5). Hence, compared with the three-term task, this group achieved less good premise retention by the end of testing.

One solution is to reduce the number of increments, whilst maintaining or increasing the number of opportunities for each child to see the premises. This could be done by spending 4 or 5 days training children in blocks lasting 15–30 minutes (Holcomb et al., 1997; cf. Riley & Trabasso, 1974). But in the current UK educational context, it is less feasible to remove a child from class for longer than one lesson period. What needed to be done then, was to somehow reduce testing, but increase training, all whilst remaining within the time limits afforded by today’s UK schools.

The second experiment addressed these issues via a new computer-based five-term extensive-training task. The aim was to give children the greatest chance of showing their transitive abilities, by removing every reasonable barrier. Crucially, as compared with the extensive-training task used in Experiment 1, twice the premise presentations were employed before a test session, whilst keeping overall test time as close as possible to that of the extensive-training task of Experiment 1. This was achieved by doubling the number of presentations in the learning phases whilst reducing the number of rounds of testing.

As well as the above two issues, which can be considered variables, there were two other issues that can be considered cohort issues. One is the possible reason that the relatively low inferential performance on the five-term task in Experiment 1 stemmed from that experiment being as inclusive as possible. Kallio (1982) found that about one-third of children tested fail pretests (essentially, tests of premise learning prior to the transitive tasks proper). Kallio noted that this exclusion practice features in much transitive research. It continues more recently, too (e.g., Whelan, Barnes-Holmes, & Dymond, 2006). In removing those children who would fail the task, the mean for the remaining group would likely be raised. Therefore, the nonuse of exclusion criteria in Experiment 1 might partly lay behind children’s relatively low performance in its five-term task.

The second cohort-based issue is that Experiment 1 ensured that children were all of similar age (range around 1.2 years for tasks combined). This contrasts with Bryant and Trabasso (1971), who ensured an age range of approximately 4 years (4 to 7 years) and reported that performance increased with age (4 years = 78%, 5 years = 84%, 6 years = 88%). It is therefore possible that including a much larger age range compared with Experiment 1 might now result in increases in performance with age. Also, as I would expect the under 7s to approach chance performance (50%), but the over 7s to approach perfect performance (100%), this should raise the overall level of performance for the group as a whole towards 75%.

Lastly, by comparing this new five-term extensive-training task to that of Experiment 1, it was possible to comment on the role of unrestricted training to previous findings with 4-year-olds.

### Method for Experiment 2

#### Participants

A total of 97 children, 5.28 to 10.00 years of age took part (*M =* 7.80 years, *SD* = 1.062). Children whose mean premise memory for the critical antecedents (BC & CD) and for the mean of the two nonantecedents (AB & DE) was not greater than chance were excluded from the analyses. These criteria led to a reduced sample of 71 children of 5.30 to 10.00 years who were included in the analyses. This sample size was very close to that of the three-term task of Experiment 1. Here, children’s mean age was 7.90 years (*SD* = 1.234), and there was an approximately equal number of boys and girls, all children having English as their first language.

#### Materials

These were similar to the five-term task of Experiment 1. The presentation regime (e.g., viewing distance) and stimuli within the new five-term extensive-training task (e.g., sizes, colours, presentation times) were similar to those of the five-term task from Experiment 1. The main departures are explained in the procedure section below. As before, a laptop PC with a 17-inch screen and an external numeric keypad were used.

#### Design

The basic design was identical to the five-term task of Experiment 1.

#### Procedure

This was the same as the five-term task of Experiment 1, notwithstanding the computer program being new. This included the provision of learning trials followed by a single test phase, as practice/familiarization trials, similar to Experiment 1. To reduce the possibility of distracting children, the need to use computer-generated curtains during learning or test trials, was dispensed with. Instead, children saw the full-length representations of items during the learning phases (explained as a side view of objects), but during test phases they saw only the tops of items (view from directly above which showed circular cross-sections of objects only, but no length information).

Next, to reduce the possibility that the use of five increments of testing in Experiment 1 had exhausted the young participants, children were tested only twice in the present experiment. However, after one initial presentation of each premise pair, they were presented with each premise a total of four times within each of the two learning phases, making for eight repetitions of each premise pair in total; twice the presentations of Experiment 1.

The first learning phase showed a premise once for 5 to 6 seconds (randomly determined), followed by a 1 to 2 second blank screen with fixation cross, followed by the next premise. Participants were instructed to carefully watch the presentation and remember which item was longer of each premise pair, but they were not required to respond to these trials. Premises were presented in random order, with one item of each premise pair on the left and the other on the right as in Experiment 1. Once all four premises had been presented once, their order was rerandomized, and then each one was presented again. Upon four presentation routines being shown, the first learning phase was complete.

A test phase followed, similar to that used in Experiment 1, except that each test trial now was presented eight times instead of four times for a more fine-grained assessment of premise learning and inferential performance. Each test trial showed a circle of each of the two colours of the items that had been seen as sticks in the learning phase. The children were told that the circles were the ends of the sticks as viewed from above. In this way, children saw a representation of each stick, but not the lengths of those sticks. Responses were given as in Experiment 1.

After the first test phase was complete, the child began the second learning phase, followed by a second test phase. Only the data from the second test phase were used. The average total time including briefing, testing, and debriefing, was 30 minutes.

### Results and discussion for Experiment 2

For the present task, each correct answer received half a mark, making the maximum possible score 4, as in Experiment 1. The bottom section of Table 1 summarizes children’s inferential and memory performance on the new five-term task against both the three-term and five-term tasks of Experiment 1. Regarding inferential performance, the last column shows that the five-term extensive-training task of Experiment 2 led to higher performance compared with the five-term extensive-training task of Experiment 1. However, it did not lead to inferential performance on the five-term extensive-training task of Experiment 2 rivalling that on the three-term task.

These tendencies were assessed using a one-way ANCOVA with the three tasks entered as levels of the variable “task”, and age and mean memory as covariates. For task, age was not a statistically significant covariate, *F*(1, 187) < 1. However, mean memory was a significant covariate, *F*(1, 187) = 11.072, *p* = .001.

With age controlled, the overall difference in inferential performance on the three tasks was statistically significant, *F*(2, 187) = 5.782, *p* = .004. Contrast analyses confirmed that the tendency for higher inferential performance on the five-term task with fewer test increments (Experiment 2), compared with on the five-term task with more test increments (Experiment 1), was statistically significant (*p* = .050). Importantly, the three-term task resulted in significantly better inferential performance than both the five-term task with less premise exposure or the task with twice the premise exposure (*p* = .001, *p* = .004, respectively).

Turning to memory, a separate two-way ANCOVA with the three tasks as levels of the between-subjects factor and the two premises as levels of the within-subjects factor confirmed a statistically significant overall difference in memory for the three respective tasks, *F*(2, 188) = 74.985, *p* < .001. As found for inference, contrast analyses showed that memory for the five-term task of Experiment 2 was significantly better than for the similar task of Experiment 1 (*p* = .001). However, memory for either of these tasks did not significantly rival memory for the three-term task of Experiment 1 (five-term task with fewer premise exposures, *p* < .001; five-term task with twice the exposures to premises, *p* < .001).

As had been the case with inferential performance, age was not a statistically significant covariate of memory performance on the three tasks taken together, *F*(1, 188) < 1. However, interestingly age was a statistically significant covariate of the difference between premise BC and CD, *F*(1, 188) = 12.673, *p* < .001. Additionally, controlling for age in this way, there was still a statistically significant difference between premise BC and CD, *F*(1, 188) = 12.208, *p* = .001. This indicated that overall, premise CD yielded reliably better performance than BC (see Table 1, which shows premise memory after controlling for age).

The ANCOVA revealed no statistically significant two-way interaction between task and individual premise retention, *F*(2, 188) < 1. From this one can infer that, although overall (i.e., mean) premise memory did differ from one task to another, the average of the BC premise and the CD premise did not reliably alter between tasks.

Thus far, the analyses have confirmed that doubling the amount of exposure to premises without increasing the time taken to train children results both in better memory and in better inferential performance. This is in line with the commonly held view that inference is always a direct by-product of premise memory (Chapman & Lindenberger, 1992; Trabasso, 1977).

Correlational and regression analyses tested the view that the major (BC) premise plays a stronger role in acquisition of the inference than the minor (CD) premise. For correlations, there were two ways of testing whether the BD inference is a direct by-product of premise memory. The first was to consider BD against each premise in turn. The second was the method used in Bryant and Trabasso (1971) of computing the product of BC and CD (i.e., BC × CD) and assessing its relationship to BD. Both methods were used in the correlational analyses. Next, observe that the difference between BC and CD covaried with children’s age. Therefore, the premise-difference score was computed to assess its correlation both with age and other variables (most notably the BD inference). The direction of this difference additionally allowed the testing of the assumption that the major BC premise takes the lead in reaching the BD inference (Bryant, 1998). As the five-term task of Experiment 1 had resulted in lowest mean premise memory and inferential performance (the latter was only 6% above chance performance), that task was not analyzed further.

The pair-wise correlations between the variables outlined here, for the three-term task and for the five-term task of Experiment 2, are presented in Table 2. Correlations of the three-term task are summarized in the top-right triangle of the table, with the five-term task summarized in the bottom-left triangle. For the three-term task, inferential BD performance significantly increased with children’s age. This is more interesting given the narrower age group of the children who completed this task. Table 2 shows that memory for the minor CD premise memory was significantly correlated with BD performance, but although the major BC premise was positively associated with BD, that correlation was not statistically significant. Both the premise-product and the premise-difference-score were significantly correlated to BD. The correlation regarding premise-difference-score was negative, indicating that smaller differences between the premises (e.g., when the child remembered both premises) were associated with higher BD performance. Finally, for the three-term task, it was interesting to note that premise retention for BC was not significantly associated with CD. This suggests that the BD inference did not derive passively from the automatic integration of the premises.

**Table 2:** Summary of bivariate correlations for two tasks in Experiment 2

	BD	Ages	BC	CD	Predict_i	Prem_Diff
BD	–	0.322 (0.002)	0.108 (0.176)	0.448 (0.001)	0.367 (0.001)	−0.242 (0.017)
Ages	−0.001 (0.498)	–	0.113 (0.165)	0.152 (0.094)	0.200 (0.040)	−0.027 (0.408)
BC	−0.221 (0.032)	−0.278 (0.009)	–	0.029 (0.402)	0.742 (0.001)	0.702 (0.001)
CD	0.487 (0.001)	0.299 (0.006)	−0.668 (0.001)	–	0.681 (0.001)	−0.692 (0.001)
Predict_i	0.281 (0.009)	−0.077 (0.262)	0.400 (0.001)	0.373 (0.001)	–	0.050 (0.334)
Prem_Diff	−0.388 (0.001)	−0.316 (0.004)	0.914 (0.001)	−0.913 (0.001)	0.016 (0.448)	–

*Note*. Top-right triangle summarises three-term task. Bottom left triangle summarises extensive-training task of Experiment 2. Figures in parentheses are levels of statistical significance. Predict_i = use product of BC & CD to predict BD; Prem_Diff = compute difference between BC & CD

The correlations regarding the five-term task of Experiment 2 contained two salient departures from that for the three-term task. The first departure was that now age did not correlate with BD performance. This finding is quite intriguing given that children were tested across a much wider age range for this task, which had been intended to maximize the likelihood that older ages were associated with higher BD performance. The second departure was that, whereas there was no association between memory for the major BC premise and the BD inference for the three-term task, for the five-term task there was a correlation, but it was in the opposite direction to the tendency for the three-term task (i.e., it was now negative). This indicates that it was children who remembered BC less well who tended to do better on the BD inference.

Concerning the sizes of correlations of BC and CD with BD, one should note that the correlation regarding CD was more than twice the magnitude of that regarding BC. Thus, CD tends to play the greater role in the BD inference. Unlike the three-term task where BC and CD were uncorrelated, for the five-term task there was a strong negative correlation between BC and CD. This indicates that children remembering one premise tended to have difficulty retaining the other premise.

Following the two correlational analyses, separate linear-regression analyses were conducted for the three-term task and five-term task. In each regression, inferential performance was entered as the criterion (dependent) variable and the two premises (BC & CD), plus age were predictors (independent) variables. Also included were the two additional variables—premise product and premise-difference-scores—as these had produced significant correlations with BD earlier (see Table 2). I opted for a step-wise hierarchical regression model to allow the statistical package (SPSS Version 24; IBM Corporation) to settle on the minimum number of variables explaining BD performance with the highest statistical reliability.

A summary of the respective regression models is given in Table 3. For the three-term task, SPSS offered two models. The final model was statistically significant and accounted for 26.7% of the variance in the inference data (*R* = .517). This model contained the CD premise and age, with each having a positive standardized beta coefficient. However, the BC premise, premise-product, and the premise-difference score were each excluded from the final model (see Table 3).

**Table 3:** Summary of regression models for two tasks in Experiment 2

	3 term	5 term
Step 1 Model *R/ R*^2^	0.448/0.201	0.487/0.237
*F* statistic	18.871 (<0. 001)	21.484 (<0.001)
Beta Coefficients		
CD	0.448 (<0.001)	0.487 (<0.001)
Step 2 Model *R/ R*^2^	0.517/0.267	
*F* statistic	6.661 (0.012)	
Beta coefficients		
CD	0.409 (<0.001)	
Ages	0.260 (0.012)	
Excluded variables		
Ages		−0.161 (0.145)
BC	0.067 (0.504)	0.189 (0.184)
Predict_i	0.069 (0.619)	0.116 (0.311)
Prem_Diff	0.093 (0.504)	0.344 (0.184)

*Note*. Figures in parentheses are levels of statistical significance. Predict_i = use product of BC & CD to predict BD; Prem_Diff = compute difference between BC & CD

In the analogous regression for the five-term task, the model was again statistically significant, accounting for 23.7% of the variance in the inference data (*R* = .487). SPSS offered only one model, and this contained only the CD premise. All other variables were excluded from the model.

## Experiment 3

The two previous experiments taken together confirmed that it is possible to obtain increased transitive performance by modifying a five-term task (Wright & Howells, 2008). It also confirmed some important differences between performance profiles on three-term versus five-term tasks.

One could object to these findings on two methodological grounds. First, the three-term task used physically present objects to imply the transitive series, whereas the five-term task presented items on computer instead of being real (physically present) objects. Thus, perhaps performance and profile differences between three-term and five-term tasks reported above were due to fewer cognitive demands associated with physical tasks versus computer-presented tasks, and not to genuine differences in transitive reasoning (my thanks to one anonymous reviewer for this observation).

A second possible objection is that in the three-term task used thus far, premise memory was assessed by using octagonal tokens to stand for the actual objects. However, in both five-term tasks, children saw the actual items both for memory and for inferential trials. Hence, the difference between tasks could have been due to the use of physical tokens in one task but not the other, instead of genuine differences. It should be noted that the use of tokens in the three-term task meant that, in addition to reasoning about the actual items in the series, the children had to mentally map the visible tokens onto representations of the objects held in memory (Wright & Dowker, 2002). If anything, this additional requirement should be expected to render the three-term task more difficult, but in Experiments 1 and 2, the three-term task led to higher, not lower performance than each five-term task (Wright, 2012). The implication is that the use of a noncomputer presentation was greater than the challenges due to the use of tokens on the above three-term task.

To address the above two possible objections, a final experiment was carried out. If the first objection is correct, then a three-term task with real objects should be easier to solve than one using computer presentation. If the second objection is correct, then removing the need for tokens should also contribute to higher performance on the three-term task having physical objects.

Experiment 3 included one additional design alteration. It was considered prudent to investigate the above prediction across a wider range of children’s age. As well as allowing direct comparison against the age groups in the Bryant and Trabasso (1971) seminal paper, this provided a test of whether physical-task versus computer-task differences might exist at some ages, but not at others. Because of working with children of younger ages than in Experiments 1 and 2 (e.g., 4–7-year-olds; Bryant, 1998), it was necessary to use fewer trials than in the earlier experiments. Thus, here, a greater number of children were tested to compensate.

### Method for Experiment 3

#### Participants

A total of 227 children between 4.26 and 7.29 years took part (*M* = 5.75 years, *SD* = 0.788). Children were typically developing, with English as their first/only language. They were allocated either to a three-term towers task or a three-term race task (see below for task descriptions). A total of 111 children, 4.26 to 7.23 years of age, were assigned to the race task (*M* = 5.76 years, *SD* = 0.723). These were divided into three groups: 37 children, 4–5 years of age (*M* = 4.96 years, *SD* = 0.276); 40 children, 5–6 years of age (*M* = 5.77 years, *SD* = 0.276); and 34 children, 6–7 years of age (*M* = 6.62 years, *SD* = 0.301). The towers task included 116 children, 4.33–7.29 years of age (*M* = 5.75 years, *SD* = 0.848). A total of 41 children, 4–5 years of age (*M* = 4.83 years, *SD* = 0.274); 38 children, 5–6 years of age (*M* = 5.76 years, *SD* = 0.251); and 37 children, 6–7 years of age (*M* = 6.77 years, *SD* = 0.341).

#### Materials

The towers task used three different sets of small, three-dimensional wooden objects, each set selecting items from a total of five available colours (red, green, blue, yellow, and grey). Two objects of each colour were contained in a box so that they were out of view of the child (as used in Wright & Dowker, 2002). The second object shape was a cylinder with a diameter of 5 cm and a height of 5.5 cm. The third objects were cuboids with a square cross-section (5 cm) and a height of 6 cm. The colours and numbers of objects for cuboids and cylinders were the same as for the cubes described above. Following Markovits, Dumas, and Malfait (1995), we also employed a white filler block, which was a 5-cm/side cube.

The race task was presented on a portable computer as in Experiments 1 and 2. This presented three cartoon children getting ready to run against each other in pairs. The children were of the same primary colours as in the towers task.

#### Design

A two factorial between-subjects design was used. Children completed one of the two tasks (towers task vs. race task), with the tasks representing one level of the first factor. The second factor was age group, with 4–5-year-olds, 5–6-year-olds, or 6–7-year-olds.

#### Procedure

Children were given one of the two task types. For the towers task, each child was shown a practice/familiarization trial with no filler block. Here, the child watched as the experimenter placed a block (B) on top of a second block (C) to form a tower of two blocks (BC). Next, a second tower containing block C above D was constructed in the same way. The experimenter told the child, “Now, I can take one of those (pointing to Block B at the top of its tower) and one of those (pointing to Block D at the bottom of its tower) and make a new tower with these two blocks. Watch me do that now.” The experimenter reached into the box of objects and retrieved a duplicate Block B and a duplicate Block D. Placing these blocks apart on the table just in front of the child, the experimenter asked, “If I look at this tower (BC) and I look at this other tower (CD) as well, do you think I should put this block (pointing to duplicate Block B) or this one (pointing to duplicate Block D) on the top of my new tower? What do you think?” After the child gave his or her answer, the experimenter constructed the new tower with Block B on top of Block D.

Although this practice trial seems transitive (Pears & Bryant, 1990), Markovits et al. (1995) observed it contains a positional cue that means the child does not have to apply deduction to get the right answer. The child can merely observe that Block B is on top of tower BC and Block D was on the bottom of the CD tower. If the child simply repeated this positioning statement for B and D when given a duplicate Block B and a duplicate Block D, he or she would arrive at the correct response, but this strategy did not entail the use of deduction. Markovits et al. therefore recommended that a filler block be placed under the CD tower, so that this nondeductive cue is neutralized. After completing the first practice trial, the child was introduced to the role of the filler block under tower CD, via one further practice trial. Here, the experimenter removed all the blocks and then placed only the white filler block on the table. The towers were then constructed as before, with the only difference being that the second (CD) tower is constructed on top of the filler block. The child was now introduced to the duplicate B and D blocks as before, and after the child gave his or her answer, the experimenter built the BD tower as before (note B and D now do not offer a height cue).

Thus, the child first gave his or her response and then witnessed the BD tower being built. However, the experimenter did not verbally give the child feedback as to whether the child’s response was correct or incorrect. Instead, the experimenter merely constructed the BD tower correctly. The child’s responses to the two practice trials were not recorded. These practice trials constituted “warm-up problems” rather than training (for first use of such trials, see Hooper, Toniolo, & Sipple, 1978).

After the two practice trials, the child completed three proper trials. In each trial, two towers representing premises BC and CD were set up about 20 cm apart on the table, and about 30 cm in front of the child. For at least one trial, BC was to the left of CD, and for the remainder of trials it was on the right (Wright & Dowker, 2002). Two duplicate blocks, one of colours B and D, respectively, were then placed between the two towers, but closer to the child—around 15 cm from the child.

One departure from the practice trials was that for proper trials the child was asked to make the third tower with the two duplicate blocks (B & D) that had been placed loose on the table in front of him or her. As the child reached forward to the blocks, the experimenter said, “Remember, first you need to tell me which one should go on top, and then you can make the tower to show me. Is that ok?” The child gave his or her response to the transitive question and then built the BD or DB tower.

One of these three trials used the cubes as items, one used cuboids and one used cylinders. The trials were given in random order across children and each trial used a different combination of colours, such that the B block was never the same colour in more than one of the three trials.

For the race task, children viewed short cartoons containing dynamically-life-like but slowed down movements of cartoon characters. These were presented on computer in full-screen mode, and were set within a three-dimensional scene displayed on the screen (Markovits & Thompson, 2008). In a practice/familiarization trial, the child watched as three boys lined up to run against each other. They were each dressed in different-coloured clothes—red, blue and green. As they ran to the finish, one boy came in first (e.g., B = red), with a different boy coming second (e.g., C = blue), and the final boy coming third (e.g., D = green). The motion sequence took around 10 seconds. The child was then shown the boys running again, with the same outcome.

Three proper trials followed. In one of these, the child watched as two girls stood on the start line. One was dressed in one colour (e.g., B = blue), and the other was dressed in a different colour (e.g., C = red). The child was told, “These two are going to run a race.” After the race, the child was then asked, “So, which one won?” After the child gave an answer, two characters again appeared at the start line, one of which was the same colour as one of the first two runners (e.g., C = red) and the other dressed in a new colour (e.g., D = green). One of these (e.g., red) crossed the finish line first.

After observing these two races, the child was shown B and D at the start line and was told that these two (e.g., blue & green) were about to run. The child was asked, “Who is going to win out of these two?” As with the towers task above, no verbal feedback was given for the transitive question on proper trials. Two further trials were given in random order, one with girls and one with boys.

Both the towers task and the race task took around 10 minutes to complete, including briefing, debriefing, instructions, and the two practice trials. Children were thanked for their participation and were returned to their classroom. For both the height task and the racing task, a child was given a mark of 1 if the answer on a trial had been correct, and 0 if incorrect. The cumulative score was taken as an estimate of transitive reasoning capacity.

### Results and discussion for Experiment 3

The maximum possible transitive (BD) score was 3. Performance according to task and age group is summarized in Table 4. The tendencies were assessed using a two-way between-subjects ANOVA, having age (three levels) and task (two levels) as factors.

**Table 4:** Transitive performance for the towers task and race task of Experiment 3

	4–5-year-olds	5–6-year-olds	6–7-year-olds	All children
Race task	1.432 (0.167) 48%	1.800 (0.161) 60%	2.056 (0.174) 69%	1.764 (0.097) 59%
Towers task	1.585 (0.159) 53%	1.684 (0.165) 56%	2.135 (0.167) 71%	1.802 (0.094) 60%
Overall tasks	1.509 (0.115) 50%	1.742 (0.115) 58%	2.097 (0.121) 70%	1.783 (0.068) 59%

*Note*. Figures in parentheses are standard errors of mean scores. Percentages are included for ease of comparison with other research

For both the three-term tasks of Experiment 3, the BD inference, overall, did not tend to differ. This was supported by a main effect of task, which was statistically nonsignificant, *F*(1, 221) < 1. However, the tendency towards higher transitive performance with age group was statistically significant, *F*(2, 221) = 6.470, *p* = .002. These two main effects taken together suggest that both these tasks were sensitive to age, but there was no advantage of the physical task (the towers task) over the computer-based task (the race task).

Post hoc comparisons using Scheffe’s test for multiple comparisons showed the improvement from 4–5 years to 5–6 years was not statistically significant (*p* = .183, one-tailed). However, the improvement from 5–6 years to 6–7 years bordered on statistical significance (*p* = .053, one-tailed), and the improvement from the youngest to the oldest age group was also significant (*p* < .001).

Consideration of the two-way interaction between task and age group would reveal whether, despite there being no overall difference between the two tasks, there was a tendency for one task to lead to higher performance relative to the other task, but this tendency tended to reverse as we move from the youngest group to the oldest group. Any such tendency, however, was nonsignificant, *F*(2, 221) < 1. This shows that neither task led to reliably higher performance for any of the three age groups. Finally, the performance of the oldest age group here was around 6% below three-term performance in Experiment 1, a tendency that is in line with the oldest group here being around 1 year younger than in Experiment 1.

## General discussion

The present findings suggest, possibly for the first time within a single empirical paper, that three-term tasks but also extensive-training tasks can validly index transitive responding. Indeed, I found several similarities between performance profiles for these two tasks as well as some intriguing differences. These are outlined below, along with consideration of what they tell us about transitive development during childhood.

First, efforts were made to ensure that a three-term nontraining task and a five-term extensive-training task were as equivalent as reasonably possible. This was achieved by using four randomized trials versus four randomized training sessions, respectively. Although premise retention on the three-term task of Experiment 1 was errorless immediately before children gave their answer to the transitive question, mean premise recall dropped to 85% immediately upon giving the transitive response. This implies that, in addition to premise memory affecting ability to make a transitive inference, the giving of a transitive response may also affect ability to retain the premises.

The premises in the five-term task were much harder to acquire than those of the three-term task. This is in line with findings from Wright (2006a/b), who estimated that adult performance when learning the 10 premises on a five-term task imposes a similar memory load as children learning only three responses on a three-term task. That said, in line with Bryant (1998), inferences were more difficult to deduce than the information they were based on in the three-term task of Experiment 1 and the five-term task of Experiment 2 (Bara et al., 2010; Markovits & Dumas, 1999; Wright & Howells, 2008; Wright & Smailes, 2015). Intriguingly, the only exception to this profile was the five-term task of Experiment 1, which was the task employing the test procedure closest to Bryant and Trabasso (1971).

The superiority of premise memory over the critical BD inference was more evident in the three-term task (contrast Breslow, 1981). Indeed, there has long been some intraparadigm disagreement regarding extensive-training task advocates (e.g., contrast Trabasso, 1977, with Bryant & Trabasso, 1971). For instance, in the Bryant and Trabasso (1971) study, the premise superiority profile is noted; but in Trabasso’s (1977) subsequent research, and many other extensive-training tasks since Trabasso, it is often reported that the inferential response is now higher than the premises on which that inference is supposed to be based (Favrel & Barrouillet, 2000; Titone et al., 2004). Trabasso himself noted the obvious answer is that when one finds an inference superiority profile (part of what he called the symbolic distance effect), one should suspect that the transitive response had not been based on deducing anything from the premises at the time of test (Trabasso, 1977; Wright & Howells, 2008). This was perhaps most clearly demonstrated in Wright (2006a), where it was found that the deductive profile did emerge during moderate training, but by the last session of testing, it had been replaced by Trabasso’s profile. The profiles across the present experiments therefore indicate that in the extensive-training task of Experiment 2, the training regime led to some degree of deduction (Trabasso et al., 1989), whereas the training regime used in Experiment 1 led to a nondeductive strategy for arriving at the BD inference (Trabasso, 1977).

Halving the number of rounds of learning and testing on a five-term task, whilst keeping the overall task duration constant, did lead to an 11% improvement in the BD inference, in relative terms. But in learning-disabled children and very young typically developing children, one can also increase transitive responding by greatly extending training instead of titrating it, regardless of whether the participants are thought to possess deductive transitive inference (Holcomb et al., 1997; Renner, Price, & Subiaul, 2016; Stromer et al., 1993). These apparently contradictory findings represent an opportunity for those seeking ways of addressing learning difficulty.

Notwithstanding this possibility, highly extensive training and memory still represent a potential issue with extensive-training tasks (Wright, 2012). Indeed, whether such tasks are even feasible within the context of children in schools today is questionable (Artman & Cahan, 1999; Castle & Needham, 2007; Rabinowitz et al., 1994). Perhaps this realization is why there seems no empirical paper based around the extensive-training task by Bryant’s lab, since the Bryant and Trabasso (1971) paper more than 4 decades ago. I suggest the extensive-training task can be of continued value in developmental reasoning research. However, future studies may need to reduce the rounds of extensive-training and testing if they wish to improve memory performance. In the present case, doing so in Experiment 2 led to premise learning some 14% better relative to the extensive-training task of Experiment 1, even though Experiment 2 did not increase overall task duration.

On the issue of rapid training, the administration of a five-term extensive-training task in less than 30 minutes is possibly the most rapidly such a task has yet been given to children (e.g., contrast Berens & Hayes, 2007; Holcomb et al., 1997; Kumaran & Ludwig, 2013; Riley & Trabasso, 1974; Van der Lely, 1997). But over and above rapidity, adopting a modified five-term task could leave researchers with sufficient time available to permit testing of young children in other domains of reasoning or social/interpersonal functioning (e.g., attentional performance, working memory, mathematical competencies, language competencies, understanding other people’s minds, attitudes regarding out groups; Birenbaum & Gutierrez, 2007; Coleman et al., 2010; Ragni & Knauff, 2013; Sedek, Piber-Dabrowska, Maio, & Von Hecker, 2011; Von Bastian & Oberauer, 2014). This was the original promise of transitive research (Binet & Simon, 1916), and it might now be realized.

Categorical effects might be an issue for extensive-training tasks. This is because one item always receives the most unmarked response or consistently positive response (e.g., is always taller in every comparison), whereas another item always receives the most marked or most consistently negative response (e.g., shorter in every comparison; Wright & Smailes, 2015). In their paper, which arguably ignited transitive research as it is conceived today, Bryant and Trabasso (1971) had advised that this issue is readily circumvented by avoiding the use of the end items (e.g., Items A and E in a five-term series). I would agree with this assertion. However, if one is not necessarily targeting a deductive transitive competence, there may be much to gain by investigating the nature of the mental representation of the entire series in memory itself, and how subsequent inferential responses are generated from it (Brunamonti et al., 2017; Kumaran & Ludwig, 2013; Qu et al., 2017; Trabasso, 1977; Van der Lely, 1997; Wright, 2006a; Wright & Howells, 2008).

Even when Experiment 2 ensured that there was much greater variability in age in the new five-term task, inferential performance still did not correlate with age, and age did not feature in its associated regression model (Castle & Needham, 2007). By contrast, for all the three-term tasks used across these experiments, increases in age or in age group were associated with increases in inferential performance. Indeed, there was little age variability in the three-term task analyzed as part of Experiment 2, yet age not only correlated with inferential performance, it also was retained alongside CD performance in the corresponding regression model (Wright, 2006b).

Thus, three-term tasks appear to relate to maturation with age as found in all experiments here, whereas five-term tasks that use extensive-training relate more to the amount of training given than to age (Brainerd & Reyna, 1992; Chapman & Lindenberger, 1992; Wright & Smailes, 2015; Wright et al., 2011). This account would explain the finding that, when the three-term task and five-term task were analyzed together in Experiment 1, age was not a significant covariate of inferential performance. This also occurred when the three-term task and the new five-term task in Experiment 2 were analyzed using ANCOVA. The explanation is that the lack of association to age on the five-term tasks had introduced enough random variability to reduce the overall reliability of the covariation, when age was a covariate across the three-term and five-term tasks analyzed together in Experiment 2.

Crucially, cognitive development is said generally to increase as age increases (Chapman & Lindenberger, 1992). Thus, if one seeks a task that will distinguish the kind of cognitive competencies that are thought to develop or mature with age, one would do better to include three-term nontraining tasks than to rely only on extensive training.

Considering the present findings, for the three-term task the two premises were retained quite independently of one another (i.e., BC did not reliably correlate with CD). However, despite this, levels of transitive reasoning were higher than for the extensive-training task regardless of amount of training given in Experiments 1 and 2. This is evidence that it is not necessary to integrate premises into a single linear array in order to achieve veridical transitive inferences (contrast Breslow, 1981; Kumaran & Ludwig, 2013).

On the other hand, for the five-term task, the two premises were now highly correlated. The fact that the correlation was negative implies that children remembering one premise tended to forget the other premise. It is as though they extracted one item of a premise (either B or D) and then used its position in the series, as an anchor from which the BD inference could be determined without an inferential strategy. For example, applying the gist that “every item to the left of D is larger” (referring to A, B, & C, respectively, but excluding Item E; Gazes et al., 2014; Brainerd & Reyna, 1992; Wright, 2001). Thus, as B would be to the left of D, assuming the child was mentally constructing the series from left to right in decreasing size, B must be the longer one: A response that would be correct even though it did not involve coordination of BC with CD in order to “deduce” BD (Breslow, 1981; Trabasso, 1977; Wright, 2012). Brainerd and Reyna (1992) observed that once the gist is generated, memory for the verbatim premises begins to fade. The present memory findings for the five-term task suggests that at least one premise is retained to enable the pivot to be constructed (Wright, 2012).

In the correlational analyses, both premises associated positively to the BD inference for the three-term task, but for the five-term task the major BC premise was negatively associated with BD. Thus, the major premise seems to feature in a different (opposite) way in the extensive-training task compared with the nontraining task. The minor CD premise and not the major BC premise featured both in the regression analysis for the three-term task and also for the five-term task (Wright & Smailes, 2015; Wright et al., 2011). Additionally, in the regressions, the BC premise did not positively relate to the BD inference, either for the three-term or the five-term task in Experiment 2. This poses difficulty for the view that the transitive series is always constructed from the large (unmarked) end (MacLean et al., 2008; Qu et al., 2017). The conclusion that it is the minor premise that pivots the series in memory is in line with data tables in several studies (Favrel & Barrouillet, 2000; Frank, Ruby, Levy, & O’Reilly, 2005; Lazareva & Wasserman, 2010; Wright, 2006a). It is also supported by data from nonhumans (Gazes et al., 2014; Treichler & Raghanti, 2010).

For the three-term task as well as the five-term tasks in Experiment 2, both the product of the premises and also the difference between the premises were correlated to the BD inference, even though in the three-term task the premises did not reliably correlate with each other. This supports the view that memory in some sense relates to inferential reasoning, at least during relatively limited premise acquisition (Chapman & Lindenberger, 1992; Gazes et al., 2014; Kumaran & Ludwig, 2013). However, Brainerd and Reyna’s (1992) findings of memory independence are not necessarily disputed. This is because, on extensive-training tasks, memory dependence can appear during premise acquisition, with memory independence replacing it if premise memory approaches asymptote (Wright, 2006a).

Perhaps computer tasks are more demanding than tasks using real objects. This could explain all the differences in profiles reported for the tasks of Experiments 1 and 2. Experiment 3 tested this prediction for three-term tasks. Here, no differences in BD performance was found, even though the two tasks used different transitive relations. One arrives at the same conclusion if previously published three-term tasks that did versus did not use computer presentations are considered (e.g., compare Ameel et al., 2007; Rabinowitz et al., 1994; with Kallio, 1988; Wright & Dowker, 2002). The objection is also ruled out for five-term tasks (e.g., compare Holcomb et al., 1997; Stromer et al., 1993; with Bryant & Trabasso, 1971; Riley & Trabasso, 1974). Therefore, the differences reported above for three-term versus five-term tasks were unlikely due to the use of real objects versus computer presentations.

Taking together, the contrasting findings, such as on premise correlation, on associations with age, and on the sign-reversal associated with the correlation between the major (BC) premise and the BD transitive inference, it would appear the two tasks tended to index two somewhat distinct ways of arriving at transitive responses (Brunamonti et al., 2017; Trabasso et al., 1989). Dual-process theory is based around two processes, sometimes labelled Type 1, or associative, versus Type 2, or analytic processing (Ameel et al., 2007; Barrouillet, 2011; De Neys & Glumicic, 2008; Evans & Stanovich, 2013; Klaczynski, 2001; Wright, 2012). Type 1 processes tend to be relatively fast, are largely unconscious, and are species general (Greene, Spellman, Dusek, Eichenbaum, & Levy, 2001; Ricco & Overton, 2011; Wright 2006a). Classical theories (e.g., Wynne, 1995) spanning transitive reasoning in humans and animals in terms of reinforcements or value transfer from one item of a premise pair (e.g., Item A) through to all other items in the transitive series (e.g., Item E), can be readily accommodated within this functional system. Type 2 processes tend to call on WM, are slower, require or result in conscious awareness, and in nonhumans these have been intimated for only a few species thus far (Kumaran & Ludwig, 2013; Libben & Titone, 2008; Premack, 2007).

However, just as systems in dual-process theory are now considered distinct but complementary (Evans & Stanovich, 2013), it is time to begin taking the transitive competencies indexed by the extensive-training and the three-term tasks as systems complementing one another rather than as mutually exclusive rivals (Frank et al., 2005; Gazes et al., 2014; Wright, 2012). Theorists can then ask new questions, such as about whether the associative mode of transitive reasoning is what gives rise to the deductive mode much later on in child development. Indeed, turning more attention to how these tasks can be used together in order to further enrich the understanding of transitive responding, plus how this relates to other aspects of social-cognition, should always have been considered the main pursuit in transitive research (Amd & Roche, 2016; Artman & Cahan, 1999; Castle & Needham, 2007; De Neys & Vanderputte, 2011; Gazes et al., 2014; Morsanyi et al., 2013; Renner et al., 2016; von Bastian & Oberauer, 2014; Wright et al., 2011).

Take learning difficulties as a case in point (Lutkus & Trabasso, 1974). Teaching techniques based on contrasts between the two types of transitive tasks can potentially help an individual follow spoken discourse embodying inferable relationships, be better able to navigate social spaces via linking together different approaches to a layout such as a town centre, be better able to make good comparisons between different items when shopping, or even better able to avoid unduly negative social comparisons between one’s self and peers (Favrel & Barrouillet, 2000; Long & Kamii, 2001; Maydak, Stromer, Mackay, & Stoddard, 1995; Rosales & Rehfeldt, 2007; Trabasso et al., 1989). This pursuit embodies the original promise of transitive research (Binet & Simon, 1916), and we are now in a better position to deliver on it to the benefit of child and learning-disabled groups than ever before.

### Conclusions

In the first direct comparison of three-term nontraining versus five-term extensive-training tasks, five-term tasks were more difficult, rather than easier to solve, as has previously been assumed. However, they became easier when highly repeated presentations of premises were given. This confirmed that training on extensive-training tasks can indeed be considered rapid if one uses double the premise exposures before the inferences are tested, instead of checking learning after every premise is presented.

Memory, in some sense, relates to inferential responses, in so far as both the product of performance on the antecedent premises and the performance difference between these premises correlate to the proportion of correct inferences. Additionally, in contrast to what has been generally assumed to date, it seems it is the minor (CD) premise that plays the more pivotal role in reaching the inference, with this the case regardless of whether one considers the extensive-training task or the three-term task.

Concerning task differences, three-term tasks were sensitive to age, regardless of whether children in a narrow age band or in a wider age band were tested. Also, the similar performance levels in the three-term tasks (using length, towers, and racing, respectively) are in line with the view that three-term tasks can index a unitary underlying transitive competence. By contrast, the two extensive-training tasks seemed insensitive to age whether I worked with a narrow or a much wider age range—and they seemed to index somewhat different competencies.

Another difference was that the premises logically necessary for deriving the transitive inference were correlated with each other on the extensive-training task, but not on the three-term task. Also, there was a tendency for the major premise (BC) to relate to the BD inference via a positive correlation for the three-term task, but via a negative correlation for the extensive-training task.

These findings are consistent with a dual-process account of transitive reasoning. Under such a conception, extensive-training tasks readily access Type 1 processes (i.e., gradually emerging associative linkages). This does not mean that these tasks never index the target deductive competence—further research is required to answer that question. Nontraining tasks more readily access Type 2 processes (i.e., willful mental simulations leading to premise coordination—deduction). If one accepts such a dual-process account for transitive reasoning, previously unsettled issues such as an extensive-training versus nontraining paradigm yielding differing estimates on age of acquisition, and on inferential difficulty, cease to be contentious. This is because each task tends to be linked to its own inferential system, with both systems essential for decision-making in the real world.
